# Synthesis and Characterization of Octacyano-Cu-Phthalocyanine

**DOI:** 10.1021/acsomega.4c04292

**Published:** 2024-07-11

**Authors:** Momoka Isobe, Fumiya Abe, Shunsuke Takagi, Kaname Kanai

**Affiliations:** Department of Physics and Astronomy, Faculty of Science and Technology, Tokyo University of Science, 2641 Yamazaki, Noda 278-8510, Chiba, Japan

## Abstract

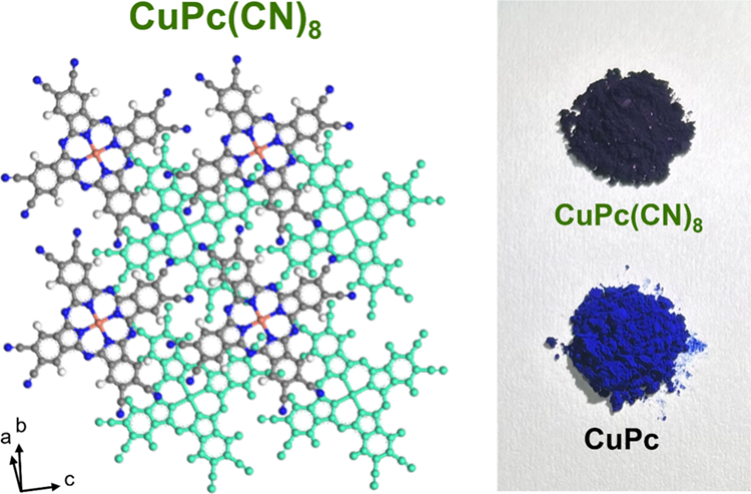

Octacyano-metal-substituted phthalocyanine MPc(CN)_8_ is a promising *n*-type stable organic semiconductor material with eight cyano groups, including a strong electron-withdrawing group at its molecular terminals. However, most MPc(CN)_8_ have not been thoroughly investigated. Therefore, CuPc(CN)_8_ was synthesized in this study and its crystal structure, chemical and electronic states, thermal stability, and electrical properties were investigated. This article discusses the various properties of CuPc(CN)_8_, as compared to those of CuPc and FePc(CN)_8_. The previously reported FePc(CN)_8_ is an organic semiconductor molecule with a molecular structure similar to that of CuPc(CN)_8_. X-ray diffraction (XRD) measurements revealed that CuPc(CN)_8_ has a crystalline structure in the *P*1̅ space group. The crystal structure forms an in-plane network parallel to the molecular plane through multiple hydrogen bonds by the cyano groups at the molecular terminals. Interestingly, the crystal structure, especially the molecular stacking, of CuPc(CN)_8_ differs from that of FePc(CN)_8_. The absorption edge observed in the ultraviolet–visible spectrum of CuPc(CN)_8_ shifted to a longer wavelength than that of CuPc, which was attributed to the energy gap of CuPc(CN)_8_ being smaller than that of CuPc owing to the influence of the cyano groups at the molecular terminals, according to the molecular orbital calculation results using density functional theory. Ultraviolet photoelectron spectroscopy measurements confirmed that CuPc(CN)_8_ had a stronger *n*-type character than CuPc because of the orbital energy stabilization by the cyano groups. Thermogravimetry/differential thermal analysis measurements revealed that the thermal stability of CuPc(CN)_8_ was significantly higher than that of FePc(CN)_8_. CuPc(CN)_8_ exhibited photoconduction upon visible-light irradiation, and its electrical conductivity was higher than that of CuPc, which was attributed to a reduction in the electron injection barrier at the electrode interfaces.

## 1 Introduction

**Figure 1:**
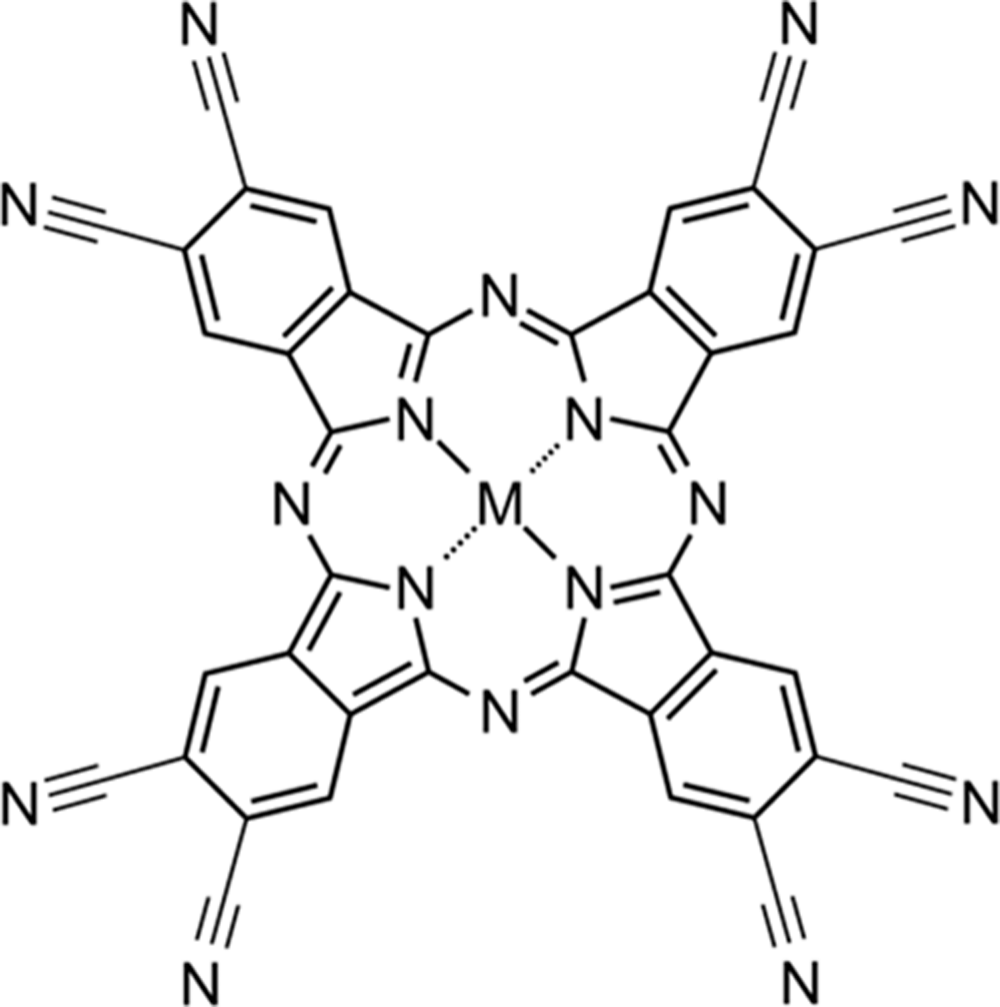
Molecular structure of octacyano-metal-substituted phthalocyanines (MPcs) (MPc(CN)_8_).

MPcs (M = Li, Mn, Fe, Co, Ni, Cu, Zn, Sn, Pb, etc.) are typical organic semiconductors and photoconductors with strong absorption in the ultraviolet–visible (UV–vis) region.^1−3^ Due to their chemical and thermal stability and the relative ease of preparing high-quality thin films, MPcs are currently being applied to a variety of devices, including solar cells, organic light-emitting diodes (LEDs), organic field-effect transistors (OFETs), gas sensors, and storage devices.^4−8^ MPcs can be functionally tuned by slight chemical modifications without losing their stability. A unique feature of MPcs is that their chemical, electrical and physical properties can be controlled by substitution of terminal groups, in addition to the type of metal substituted. For example, many MPcs exhibit *p*-type electrical conductivity,^3,9,10^ which can be easily switched to *n*-type conductivity by adding electron-withdrawing groups. Perfluoro MPcs (F_16_MPcs), in which all terminal hydrogens are substituted with fluorine, are well-known to have high electron affinity. This is because the strong electronegativity of fluorine lowers the molecular orbital (MO) energy of the phthalocyanine molecule. F_16_MPc is a typical *n*-type organic semiconductor that is chemically stable in air and has been used in *n*-type OFET applications.^6,11,12^ Octacyano-MPc (MPc(CN)_8_s) (Figure 1) has eight high-electronegativity cyano groups as the terminal groups, making it an *n*-type substance with a large electronegativity similar to F_16_MPcs. Octacyanotetrapyrazinoporphyrazine has also been reported as a molecule with very strong electronegativity containing cyano groups at the molecular terminals, similar to MPc(CN)_8_.^13^ MPc(CN)_8_ is also known as an intermediate in the thermal polymerization of poly MPc (MPc-MOF), a two-dimensional (2D) polymerized MPc framework.^14−16^ The heating of MPc(CN)_8_ during the polymerization process causes the cyano groups at the terminals of different MPc(CN)_8_ molecules, which are the binding sites, to react, forming a two-dimensional polymer layer. MPc-MOFs are rapidly gaining attention, with recent theoretical studies pointing to their potential to become semiconductors with narrow energy gaps and new magnetic materials.^17^ While many existing two-dimensional materials, such as graphene, are nonmagnetic, several MPc-MOF containing transition metals are theoretically predicted to exhibit ferromagnetic or antiferromagnetic properties, making them particularly promising new magnetic materials.^17^ Although the synthesis of MPc(CN)_8_ has been studied,^15,18,19^ the detailed chemical and physical properties of MPc(CN)_8_ have not yet been reported, except for a recent detailed report on FePc(CN)_8_.^20^ Because of the chemical instability of organic anion radicals, the options for *n*-type materials in organic semiconductors are generally limited. Therefore, understanding the basic properties of MPc(CN)_8_ should provide important insights into the design of new stable *n*-type molecules and their application in organic optoelectronic devices. Furthermore, the establishment of a simple synthesis method for MPc(CN)_8_ will significantly aid research on MPc-MOFs.

This article reports the synthesis and detailed physical and chemical properties of CuPc(CN)_8_. First, the crystal structure of the product obtained by the calcination of 1,2,4,5-tetracyanobenzene (TCNB) and CuCl_2_ was determined using X-ray diffraction (XRD), and its chemical state was investigated using Fourier transform infrared spectroscopy (FTIR) and X-ray photoelectron spectroscopy (XPS). The results show that CuPc(CN)_8_ can be obtained as a product under the appropriate synthesis conditions. Thermogravimetry/differential thermal analysis (TG-DTA) measurements revealed that the thermal stability of CuPc(CN)_8_ is significantly better than that of FePc(CN)_8_. The electronic states near the frontier orbitals were also revealed using ultraviolet photoelectron spectroscopy (UPS), density functional theory (DFT) MO calculations, and UV–vis absorption spectroscopy. The frontier orbitals in CuPc(CN)_8_, as compared with those in CuPc, were stabilized by the introduction of cyano groups and exhibited large ionic energies and electron affinities. The electrical conductivity of CuPc(CN)_8_ was significantly higher than that of CuPc. Moreover, CuPc(CN)_8_ also exhibited photoconductivity.

## 2 Experimental Section

### 2.1 Preparation of CuPc(CN)_8_

CuPc(CN)_8_ was synthesized by weighing TCNB (Tokyo Chemical Industry Co. (TCI), T0988–5G, purity: >98%) and CuCl_2_ (Merck KGaA, 8.18247.0100, assay: ≥98.0%) to a total weight of 0.2 g at a molar ratio of 2:1 and carefully grinding and mixing them using a mortar. The mixed powder was placed in a glass test tube ozonated for 15 min, and the inside of the test tube was evacuated to a pressure of *P* < 10° Pa using a rotary pump. The glass ampules were sealed, wrapped in aluminum foil, and placed in a box furnace (AS ONE, MMF-1) for calcination. The calcination process involved heating the ampules to 300 °C at 5 °C/min, holding at 300 °C for 10 h, followed by naturally cooling them to room temperature (∼20 °C). After calcination, the ampules were opened to obtain a dark blue solid. The obtained solid was crushed into a powder using a mortar and washed with pure water (FUJIFILM Wako Pure Chemical Corporation, 161–08247) and ethanol (FUJIFILM Wako Pure Chemical Corporation, 057–00451, assay: 99.5+%) to remove the unreacted CuCl_2_. The obtained sample was placed in a desiccator and vacuum-dried overnight using a diaphragm pump to remove the solvent.

FePc(CN)_8_ was prepared according to the method described in the literature.^20^

### 2.2 Characterization

The XRD patterns of the powder were recorded using a diffractometer (Rigaku, Ultima IV) equipped with a Cu–Kα radiation source. The FTIR spectra of samples embedded in KBr pellets were acquired using a spectrometer (JASCO Corporation, FTIR-6100). XPS (JPS-9030/JEOL Ltd.) measurements were performed using Al Kα radiation (λ = 1486.6 eV) as the excitation source. The XPS profiles were analyzed using Voigt functions with XPSPEAK41 software (written by Raymund W. M. Kwok). Electron spin resonance (ESR) measurements were performed using a Bruker EMX-Nano instrument. Measurements were performed at room temperature (∼20 °C) using microwaves at the X-band frequency. The samples were measured using NMR 5 mm-sample tubes (NES-600/OPTIMA). TG-DTA was conducted using a TG-DTA 2010SA instrument (Bruker AXS). TG and DTA curves were acquired in a dry N_2_ atmosphere at a heating rate of 5 °C/min. UV–vis absorption spectra were acquired using a spectrometer (UV-1800, Shimadzu Corporation). The samples (CuPc (α-form; TCI, P1005–25G, purity: >90.0%) or CuPc(CN)_8_) were dissolved in tetrahydrofuran (THF) (FUJIFILM Wako Pure Chemical Corporation, 200–00486, assay: 99.5%+) and the solution was placed in a quartz cell.

UPS measurements were performed under ultrahigh vacuum at a base pressure of 4.0 × 10^–8^ Pa using an electron analyzer (SES200, Scienta) and helium discharge lamp. The UPS spectra were acquired using the He Iα resonance line (*h*ν = 21.22 eV) as the excitation source. The Fermi level (*E*_F_) was determined from the Fermi edge of the gold substrate. CuPc(CN)_8_ cannot be prepared via vacuum deposition owing to thermal decomposition upon heating. Consequently, the CuPc(CN)_8_ samples used for the UPS measurements were prepared by the dropwise addition of 10 μL chlorobenzene (FUJIFILM Wako Pure Chemical Corporation, 032–07986, assay: 99.0+%) to the gold substrate, followed by the addition of 40 μL of a saturated *N*,*N*-Dimethylacetamide (FUJIFILM Wako Pure Chemical Corporation, 042–18656, assay: 97.0%+) solution of CuPc(CN)_8_. The samples were dried overnight under vacuum using a diaphragm pump and then dried again under vacuum using a diaphragm pump for approximately 1 min after the addition of a drop of acetone (FUJIFILM Wako Pure Chemical Corporation, 014–00347, purity: >99.5%). The gold substrates were obtained by sputtering chromium (20 nm) onto Si(100) wafers, followed by the deposition of gold (200 nm) using sputtering. The prepared gold substrates were cleaned with UV-ozone for 10 min immediately prior to use. The CuPc samples were thin films prepared by vacuum evaporation on gold substrates. The CuPc film thickness, which was measured using a quartz crystal microbalance, was 10 nm. The gold substrates were then obtained by the vacuum evaporation of gold on Si(100) wafers. Electric current measurements were performed using a source-measure unit (6487 J, Keithley) and direct current power source (R6144, Advantest).

### 2.3 Theoretical Calculations

The XRD profiles were calculated using reflex/powder diffraction in the BIOVIA Materials Studio software. The geometry of the CuPc(CN)_8_ crystal was optimized using CASTEP, BIOVIA Materials Studio with the GGA/Perdew–Burke–Ernzerhof functional and pseudopotentials: OTFG ultrasoft. The FTIR simulations were performed for a single molecule using Gaussian09 (B3LYP/6-31G(d)). The optical spectra were simulated using time-dependent DFT (TD-DFT) with Gaussian09 (B3LYP/6-31G(d)). DFT-based MO calculations for the isolated molecules were performed using Gaussian09 (B3LYP/6-31G(d)). The simulated UPS spectra were obtained by broadening the calculated MOs using the Voigt function to reproduce the observed spectra. The *S* = 1/2 doublet ground states of CuPc and CuPc(CN)_8_ were theoretically calculated.

## 3 Results and Discussion

### 3.1 Crystal Structure of CuPc(CN)_8_

**Figure 2:**
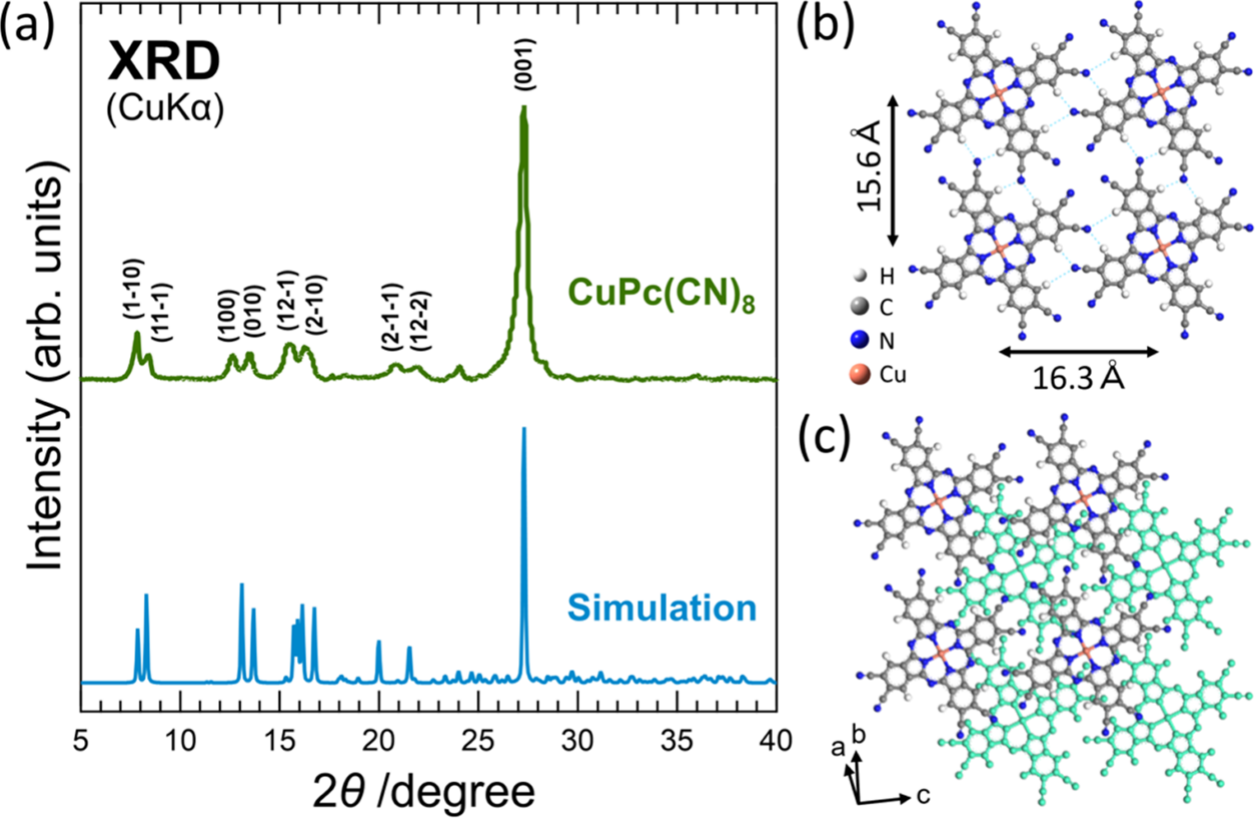
(a) XRD patterns of CuPc(CN)_8_. The lower part of the graph shows the simulated diffraction pattern. The CuPc(CN)_8_ crystal structures obtained by analyzing the XRD results are depicted in (b, c). (b) Molecular layer formed by hydrogen bonds between the terminal cyano groups. Adjacent CuPc(CN)_8_ molecules in the molecular layer are bound through multiple hydrogen bonds. The light-blue dashed lines indicate hydrogen bonds. (c) Crystal structure viewed perpendicular to the molecular layer. The green molecular layer is stacked on top of the molecular layer in (b).

Figure 2(a) shows the powder XRD and simulated diffraction patterns of CuPc(CN)_8_. Figure 2(b),2(c) show the crystal structures obtained from the analysis of the XRD results. The simulated pattern corresponded well with the measured data, although the intensity ratios of (1–10) and (11–1) slightly differed. A comparison of the experimental XRD patterns with the simulated patterns indicated that CuPc(CN)_8_ also forms hydrogen bonds between the nitrogen atoms of the cyano group at the molecular terminal and hydrogen atoms of the adjacent CuPc(CN)_8_ molecules, as previously reported for FePc(CN)_8_.^20^ However, the stacking of CuPc(CN)_8_ differed from that of FePc(CN)_8_. FePc(CN)_8_ has an x-form crystal structure where the FePc(CN)_8_ molecules are stacked on top of each other with their molecular planes and the iron ions at the molecular centers are arranged in a one-dimensional linear chain.^20^ However, the copper ions of the underlying molecules of CuPc(CN)_8_ are located and stacked directly below the voids where the cyano groups of the four molecules protrude in the molecular layer. The simulation results indicated that CuPc(CN)_8_ belongs to a triclinic system in the P1̅ space group with the lattice constants of *a* = 7.1477 nm, *b* = 10.663 nm, and *c* = 11.555 nm and unit cell angles of α = 90.3353°, β = 76.7172°, and γ = 86.8458°. Table S1 lists the atomic coordinates. The interlayer distance was 3.25 Å, which is 0.02 Å shorter than that of FePc(CN)_8_.^20^ The crystal structure of CuPc(CN)_8_-Cu on a copper substrate synthesized in a previous study using chemical vapor deposition exhibited an x-form crystal structure (*P*4/*mcc*) similar to that of FePc(CN)_8_, which differs from that synthesized in this study.^21−23^ CuPc(CN)_8_-Cu is a compound in which a CuPc(CN)_8_ and copper complex are stacked on a copper substrate. A previous study reported the synthesis of a powdered CuPc(CN)_8_ sample; however, to the best of our knowledge, its crystal structure has not yet been determined.^15^

### 3.2 Chemical and Electronic States of CuPc(CN)_8_

**Figure 3:**
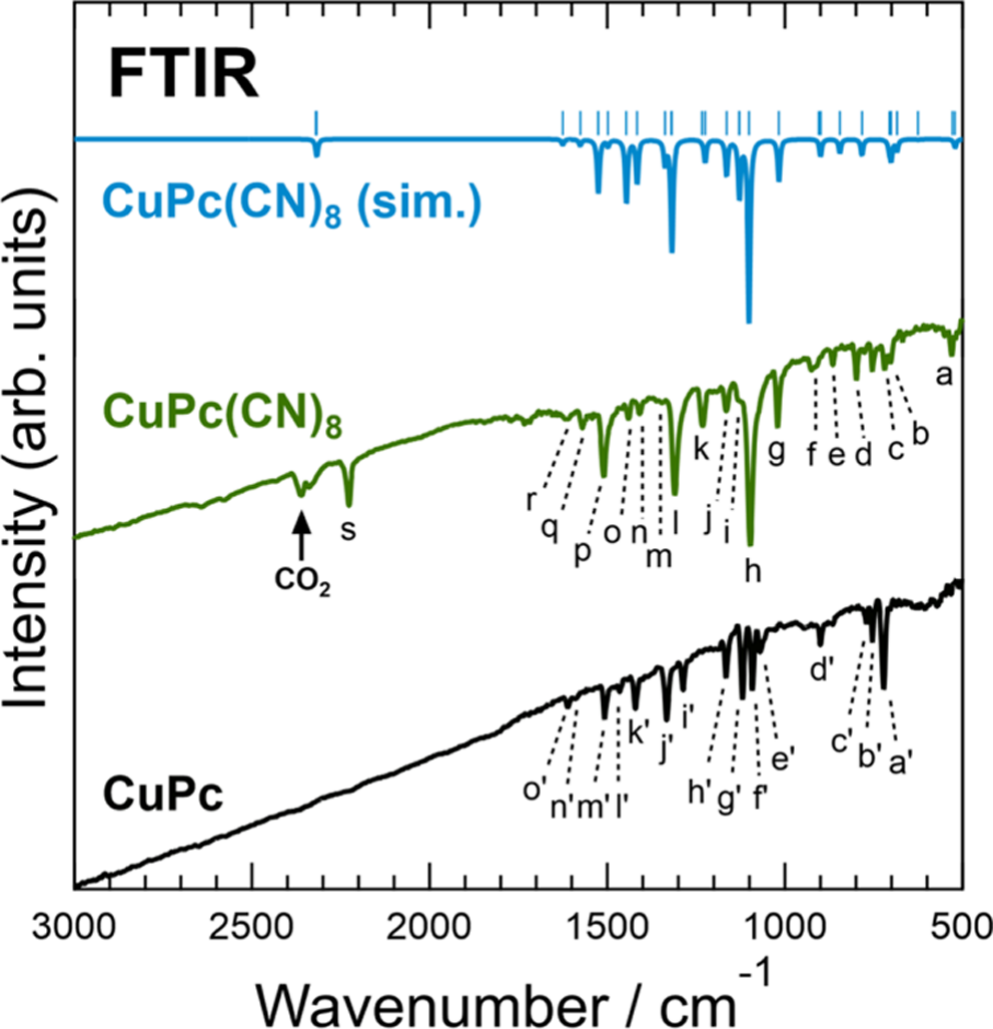
FTIR spectra of the CuPc(CN)_8_ and CuPc samples. The labels on each peak correspond to the vibrations listed in Tables 1 (CuPc(CN)_8_) and 2 (CuPc). The upper part of the graph shows the simulated FTIR results for a single CuPc(CN)_8_ molecule. The simulated spectra were shifted by approximately −34 cm^–1^ to reproduce the peak positions in the fingerprint region of the measurements. This slight discrepancy originates from the improper incorporation of the anharmonic term in the vibration calculation.

FTIR measurements provide information on the types of chemical bonds and functional groups present in a sample, providing insights into its molecular structure. Figure 3 shows the FTIR spectra of CuPc(CN)_8_ and CuPc. Tables 1 and 2 list the detailed assignments of the vibrational peaks for CuPc(CN)_8_ and CuPc, respectively. The peak at approximately 2350 cm^–1^ in the CuPc(CN)_8_ spectrum was caused by the atmospheric CO_2_, which could not be removed by background subtraction. The peak at 2227.4 cm^–1^ in the CuPc(CN)_8_ spectrum (labeled as “s”), which was not observed in the CuPc spectrum, is an absorption peak owing to the stretching vibration ν(C≡N) of the cyano group. As shown in Figure 2(b),2(c), the CuPc(CN)_8_ crystal structure in the molecular layer was organized by hydrogen bonds between the molecules. Therefore, the intermolecular hydrogen bond softened the stretching vibrations of the cyano groups, and the observed ν(C≡N) peak position shifted to a lower wavenumber by approximately 90 cm^–1^, as compared to the simulation results for a single CuPc(CN)_8_ molecule.^24^ The spectra in the fingerprint regions of CuPc(CN)_8_ and CuPc exhibited shapes characteristic of MPc. Therefore, it was confirmed that the CuPc(CN)_8_ synthesized in this study had a phthalocyanine backbone with cyano groups. Previous studies on the synthesis of FePc(CN)_8_ and CuPc(CN)_8_-Cu reported that a large C=O stretching vibration ν(C=O) peak appeared at approximately 1700 cm^–1^ due to the hydrolysis of the cyano groups at the FePc(CN)_8_ molecular terminals.^20,23^ However, the CuPc(CN)_8_ synthesized in this study exhibited almost no peak at approximately 1700 cm^–1^. This is because the amount of moisture absorbed by the CuCl_2_ used as the raw material for CuPc(CN)_8_ was adjusted to be small. The observed FTIR spectra of CuPc(CN)_8_ corresponded well with the simulation results, with several peaks originating from impurities. Therefore, a powdered CuPc(CN)_8_ sample with a smaller amount of impurities hydrolyzed from the cyano groups at the molecular terminals, as compared to FePc(CN)_8_, was successfully synthesized in this study.

**Table 1:** Assignment of the CuPc(CN)_8_ FTIR Vibrational Peaks^a^

	Exp./cm^–1^	Calc./cm^–1^	assignments
a	532.26	521.32	δ_out_(C–C≡N), δ_out_(C–H)
b	703.89	684.34	δ_in_(isoindole)
c	719.32	700.58	δ_in_(benzene)
-	754.03	-	-
d	799.35	782.91	δ_in_(isoindole)
e	864.92	845.48	ν(pyrrole C–N=C C=C)
f	920.84	899.31	δ_out_(C–H)
g	1020.2	1017.3	ν(isoindole C=C), δ_in_(benzene)
h	1097.3	1100.8	δ_in_(C–H), ν(pyrrole C–N=C)
i	1130.1	1128.2	ν(pyrrole C–N=C), δ(benzene), δ_in_(C–H)
j	1164.8	1164.1	δ_in_(benzene)
k	1231.3	1224.7	ν(isoindole benzene C=C)
l	1310.4	1318.2	ν(isoindole benzene C=C)
m	1343.2	1337.7	ν(Cu–N)
n	1408.7	1416.2	ν(N–C=N), ν(isoindole benzene C=C), δ_in_(C-H)
o	1440.6	1446.1	ν(isoindole C=C), δ_in_(C–H)
-	1490.7	1497.5	ν(pyrrole C–N=C), δ_in_(C–H)
p	1509.0	1525.4	δ_in_(pyrrole C–N=C)
q	1570.7	1576.0	ν(benzene C=C)
r	1613.2	1625.7	ν(isoindole benzene C=C), δ_in_(C–H)
-	1723.1	-	-
-	1770.3	-	-
s	2227.4	2317.8	ν(C≡N)

a The experimental (Exp.) and calculated (Calc.) wavenumbers observed in Figure 3 are summarized. ν and δ represent the stretching and angular vibrations, respectively. δ_in_ and δ_out_ represent the in-plane and out-of-plane angular vibrations, respectively. The simulated spectra were shifted by approximately −34 cm^–1^ to better explain the observed absorption peaks in the fingerprint region in Figure 3.

**Table 2:** Assignment of the CuPc FTIR Vibrational Peaks^a^3

	Exp./cm^–1^	Calc./cm^–1^	assignments
-	-	553.44	δ_in_(isoindole)
-	-	621.97	δ_in_(isoindole)
a′	722.21	699.69	δ_out_(pyrrole C–N=C)
b′	754.03	738.48	δ_in_(isoindole)
c′	770.42	754.63	δ_out_(C–H)
d′	899.63	886.15	δ_in_(C–N=C), δ_in_(benzene)
e′	1070.3	1063.3	δ_in_(isoindole)
f′	1091.5	1100.5	ν(pyrrole C–N=C), δ_in_(C–H)
g′	1119.5	1119.6	δ_in_(C–H)
h′	1165.8	1169.5	δ_in_(C–H)
i′	1286.3	1297.0	δ_in_(C–H)
j′	1332.6	1355.4	ν(benzene C=C)
k′	1421.3	1433.5	δ_in_(pyrrole C–N=C), δ_in_(C–H)
l′	1464.7	1487.2	δ_in_(C–H), ν(benzene C=C)
m′	1507.1	1527.0	δ_in_(pyrrole C–N=C)
n′	1589.1	1610.5	ν(benzene C=C)
o′	1611.2	1631.9	ν(benzene C=C)

a The wavenumbers observed in Figure 3 (Exp.) and those obtained by calculation (Calc.) in Figure S1 are summarized. ν and δ represent the stretching and angular vibrations, respectively. δ_in_ and δ_out_ represent the in-plane and out-of-plane angular vibrations, respectively. For the assignment of each peak, the simulated spectrum was shifted by approximately −31 cm^–1^ to better explain the observed absorption peaks in the fingerprint region in Figure 3.

**Figure 4:**
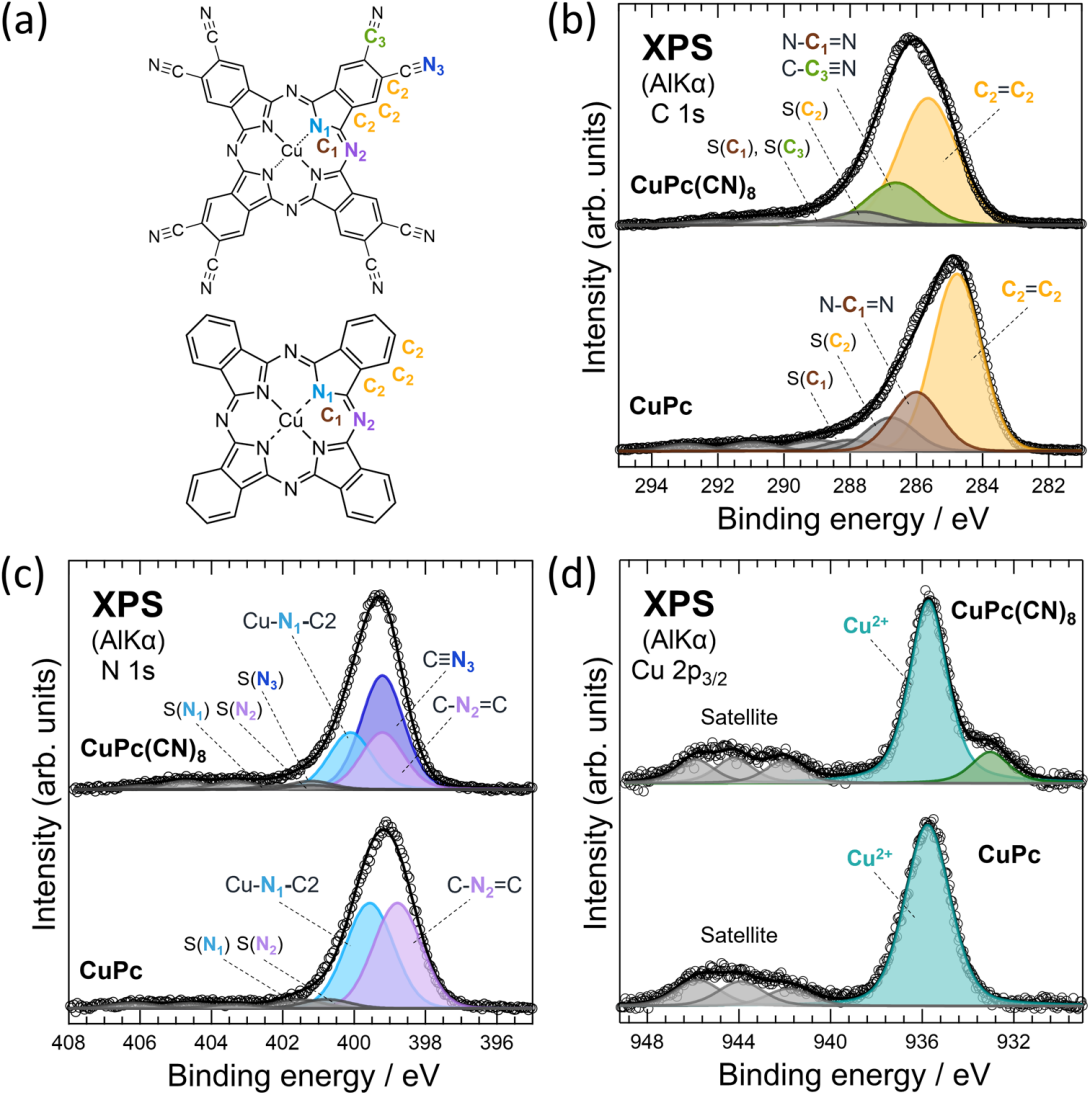
(a) Molecular structures of CuPc(CN)_8_ and CuPc. The colors of the elements in the figure correspond to the respective peaks obtained by the standard fitting analysis of the XPS spectra in (b, c). (b) XPS results of the C 1s core levels of CuPc(CN)_8_ and CuPc. (c) XPS results of the N 1s core levels of CuPc(CN)_8_ and CuPc. (d) XPS results of the Cu 2p_3/2_ core levels of CuPc(CN)_8_ and CuPc. Circles in the figure represent the data after subtracting the background from the raw data, which was determined by fitting analyses with the Shirly type background. Black lines in (b–d) represent the peak fitting analysis results. Full widths at half-maximum of Voigt functions used for the fitting analysis were 2.09 eV (C 1s of CuPc(CN)_8_), 1.73 eV (C 1s of CuPc), 1.42 eV (CuPc(CN)_8_ of N 1s), 1.59 eV (CuPc of N 1s), 1.96 eV (Cu 2p_3/2_ of CuPc(CN)_8_), and 2.34 eV (Cu 2p_3/2_ of CuPc).

The relative intensity ratios of the contributions from each element in the core-level spectra obtained from the XPS spectra and the binding energies of each peak provide information on the chemical composition and chemical state of the sample. Figure 4(b) shows the C 1s XPS profiles of CuPc(CN)_8_ and CuPc. Considering that CuPc(CN)_8_ and CuPc contain three and two carbons in different chemical environments, respectively, fitting analyses were performed with the internal intensity ratios of C_1_:C_2_:C_3_ = 1:3:1 for CuPc(CN)_8_ and C_1_:C_2_ = 1:3 for CuPc. In addition, the fitting analyses for C 1s XPS results were performed by taking both contributions from main peaks and satellite peaks into account.^25^ The fitting results are summarized in Table S2. It is noted that the contribution from the carbon bonded to oxygen in C–C=O assuming the presence of impurities in the sample formed by the hydrolysis of the CuPc(CN)_8_ molecular terminals was not necessary to reproduce the spectrum, indicating that the intensity of C–C=O in the CuPc(CN)_8_ spectrum was considerably smaller than that of the previously reported FePc(CN)_8_.^20^ The XPS survey scan in Figure S2 also confirmed the low oxygen content in the CuPc(CN)_8_ sample (O 1s XPS result is given in Figure S3). Although CuPc(CN)_8_ and FePc(CN)_8_ were synthesized using the same calcination process, the reason for the former containing almost no impurities resulting from the hydrolysis of cyano groups is still unclear; however, it is assumed that this may be due to the CuCl_2_ raw material containing less moisture than the FeCl_2_ raw material. A fitting analysis of the N 1s XPS spectra of CuPc(CN)_8_ and CuPc shown in Figure 4(c) was performed considering three and two nitrogen contributions in different chemical environments, respectively. This fitting analysis explained the observed spectra with stoichiometric internal intensity ratios of N_1_:N_2_:N_3_ = 1:1:2 for CuPc(CN)_8_ and N_1_:N_2_ = 1:1 for CuPc. The fitting analyses for N 1s XPS results were performed by taking both contributions from main peaks and satellite peaks into account.

The abundance ratios of C/N, N/Cu, and C/Cu of CuPc(CN)_8_ calculated using the XPS results were 3.3, 13, and 44, respectively, which are close to the values of 2.5, 16, and 40, respectively, which were calculated using the stoichiometric ratios. Furthermore, the C 1s and N 1s spectra of CuPc(CN)_8_ were shifted to higher binding energies than those of CuPc. This result is similar to those of FePc(CN)_8_ and FePc, and originates from the depletion of the electron density at the molecular center of CuPc(CN)_8_ because of the cyano groups at the molecular terminals.

Figure 4(d) shows the Cu 2p_3/2_ XPS spectra of CuPc(CN)_8_ and CuPc. The blue peaks correspond to the main Cu^2+^ peak. The intense satellite peaks at 940–948 eV are charge-transfer-multiplet satellites that appear only for Cu^2+^.^26^ Therefore, it was confirmed that the synthesized CuPc(CN)_8_ contains Cu^2+^. The relative intensities of the main peak and charge-transfer-multiplet satellite, as well as the energy difference between the main and satellite peaks, vary depending on the covalent bond character between copper and the ligands.^26^ The energy difference between the main and satellite peaks of Cu^2+^ and their intensity ratios in CuPc(CN)_8_ and CuPc were approximately consistent, indicating that the Cu^2+^ in the CuPc(CN)_8_ sample is coordinated to the phthalocyanine backbone. The shoulder structure at 933.0 eV in the CuPc(CN)_8_ spectrum is considered to be a zerovalent Cu (Cu(0)) peak judging from its binding energy. Cu(0) content is about 0.2% of the constituent elements of the sample. This indicates that copper nanoparticles were deposited on the sample surface during thermal polymerization. However, the absence of metallic copper peaks in the XRD spectra may be due to a very small contribution from the surface region during XRD, in contrast to XPS, which is very surface-sensitive.

**Figure 5:**
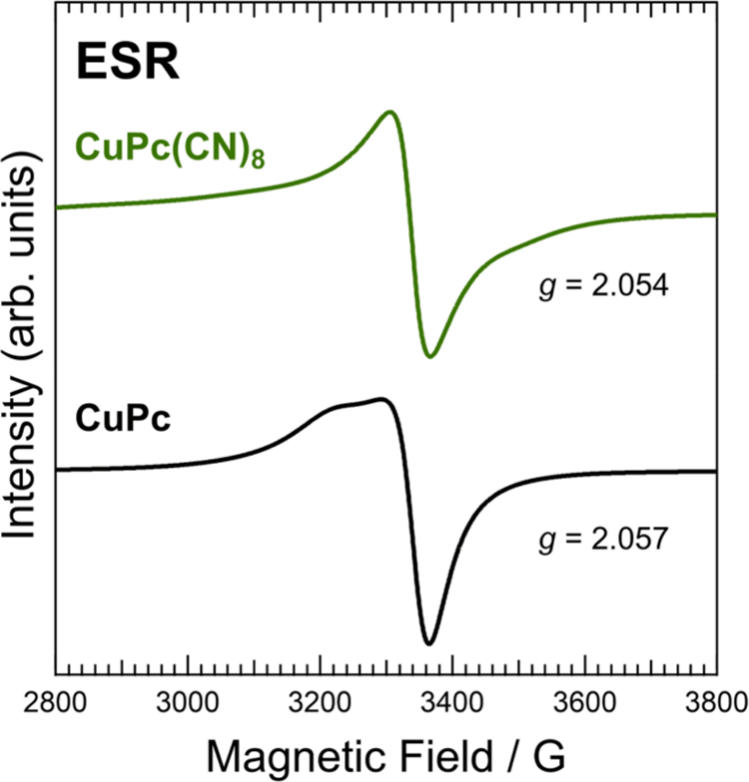
ESR results for CuPc(CN)_8_ and CuPc. The *g*-values were obtained using the *g*-value of the standard MgO:Cr^3+^ sample.

ESR measurements detect unpaired electrons in a sample and provide information on the MOs occupied by the unpaired electrons. Figure 5 shows the ESR spectra of the CuPc(CN)_8_ and CuPc powder samples. Both CuPc(CN)_8_ and CuPc were ESR-active and clear spectra were acquired. The shape of the CuPc ESR spectrum was consistent with that reported in previous studies.^27−29^

ESR was conducted on the CuPc(CN)_8_ and CuPc powder samples without dilution with diamagnetic or other materials; therefore, the hyperfine structures appearing in the spectra were averaged out because of dipole–dipole and exchange interactions.^27,28^ The CuPc spectrum exhibited a largely asymmetric shape, which was attributed to the anisotropy of hyperfine interactions.^27,28^Figure 5 shows that the CuPc(CN)_8_ spectrum has a narrower line width than that of CuPc. F_16_CuPc, which has fluorines with high electronegativity at the molecular terminals, also exhibits narrower ESR spectra than CuPc,^29^ possibly because of the restriction of the exchange interaction and the suppressed molecular vibrations of the nitrogen in the isoindole and Cu^2+^.^29,30^ A similar phenomenon should occur in CuPc(CN)_8_ with cyano groups with large electronegativity and fluorines.

**Figure 6:**
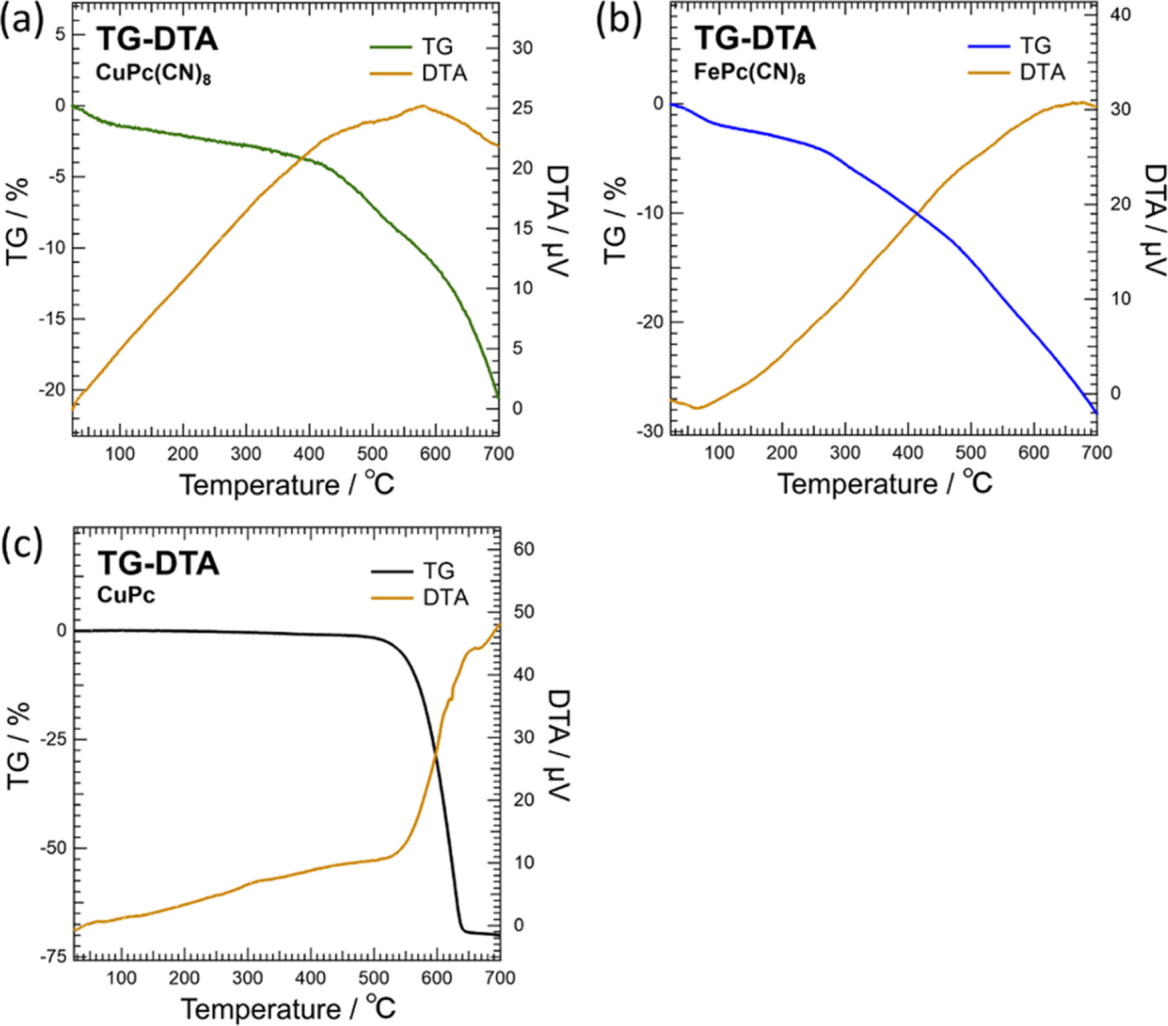
TG-DTA measurements of (a) CuPc(CN)_8_, (b) FePc(CN)_8_, and (c) CuPc.

The thermal stability of CuPc(CN)_8_ was considerably better than that of FePc(CN)_8_. The TG-DTA results for CuPc(CN)_8_, FePc(CN)_8_, and CuPc shown in Figure 6 indicate that both CuPc(CN)_8_ and FePc(CN)_8_ exhibited different mass-loss behaviors in the four temperature regions. Although the CuPc mass remained almost constant up to approximately 500 °C, CuPc(CN)_8_ and FePc(CN)_8_ exhibited relatively rapid mass losses up to approximately 100 °C and gradual mass losses at approximately 100–420 and 100–260 °C, respectively. The cyano groups of CuPc(CN)_8_ and FePc(CN)_8_ are also likely to form hydrogen bonds with the water molecules present at the grain boundaries. The rapid mass loss below 100 °C was attributed to the desorption of water on the surface of the crystal grains, and that above 100 °C was attributed to the gradual desorption of water between the crystal grains. The TG temperature variation of CuPc(CN)_8_ and FePc(CN)_8_ showed that after a gradual mass loss above 100 °C, the degree of loss increased from approximately 420 and 260 °C, respectively. The thermal decomposition of CuPc(CN)_8_ and FePc(CN)_8_ began near the temperature at which the degree of mass loss increased, and the mass loss increased with the desorption of the decomposed material. CuPc(CN)_8_ started to decompose at approximately 420 °C. This study experimentally confirmed that at least some of CuPc(CN)_8_ did not decompose even after heating to 470 °C. The FTIR spectrum acquired after heating CuPc(CN)_8_ at 470 °C for 24 h in a nitrogen atmosphere at a pressure of 1.9 × 10^2^ Pa was almost identical to that of the sample before heating (Figure S4). This indicates that CuPc(CN)_8_ does not entirely thermally decompose above a certain temperature; rather, the decomposition proceeds gradually. Therefore, it was confirmed that CuPc(CN)_8_ is difficult to decompose even at temperatures higher than 400 °C. The above results revealed that CuPc(CN)_8_ has a thermal stability approximately 160 °C higher than that of FePc(CN)_8_, which has the same molecular structure except for the metal at the molecular center. The above results demonstrate that CuPc(CN)_8_ was successfully synthesized in this study.

### 3.3 Electronic Structure of CuPc(CN)_8_

**Figure 7:**
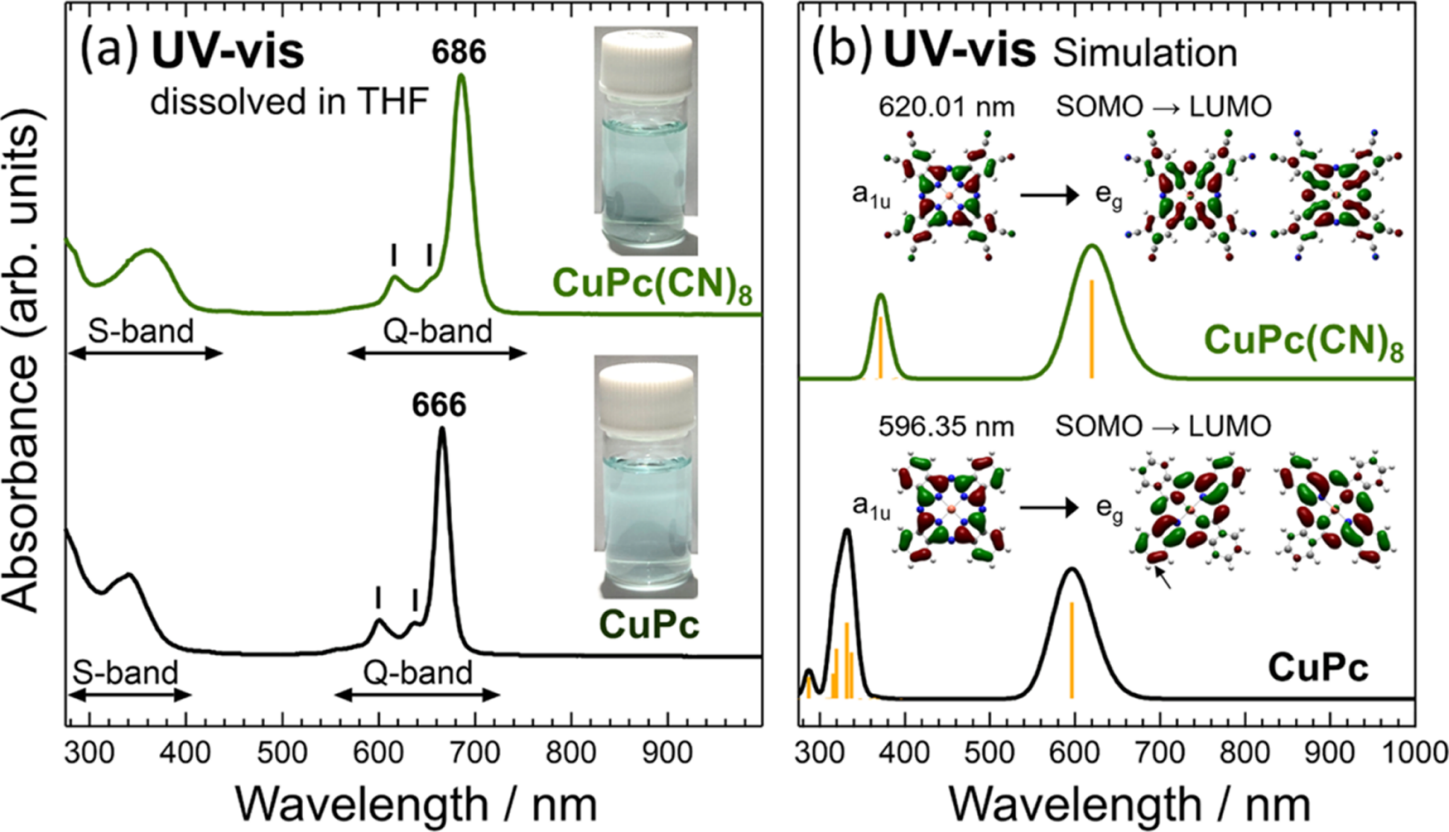
(a) UV–vis spectra of CuPc(CN)_8_ and CuPc dissolved in THF. Inset photographs show the THF solutions of each sample. The short black vertical lines above the spectra represent vibrational transitions. (b) Simulated UV–vis spectra of CuPc(CN)_8_ and CuPc. The spectra shown in green and black are the spectra broadened with a Gaussian function with a half-width at half-maximum of 0.1 eV. The MOs and transition energies shown in the figure were obtained using DFT calculations. Red and green lobes indicate the different signs of the MOs. SOMO and LUMO are abbreviations for the singly occupied molecular orbital and the lowest unoccupied molecular orbital, respectively.

Figure 7(a) shows the UV–vis spectra of the CuPc(CN)_8_ and CuPc THF solutions. UV–vis spectra provide information on the electronic structure near the energy gap, including the frontier orbitals of the molecule. The two large absorption bands in the CuPc(CN)_8_ and CuPc spectra labeled as the S- and Q-bands are characteristic of phthalocyanines. The Q-band exhibits a single large peak in both spectra, indicating that both molecules are phthalocyanines with *D*_4*h*_ symmetry and metal coordination at the molecular center. The CuPc(CN)_8_ and CuPc peaks at 686 and 666 nm, respectively, which are indicated by the short black vertical lines to the left of the main peak of the Q-band, are caused by absorption through vibrational transitions.^31,32^ The CuPc(CN)_8_ and CuPc spectra are very similar; however, the CuPc(CN)_8_ spectrum, as compared to that of CuPc, is red-shifted by approximately 20 nm. Although the degree of the red shift was smaller than that of FePc(CN)_8_ (red-shift of approximately 30 nm, as compared to FePc),^20^ it was confirmed that the addition of cyano groups at the molecular terminals caused a similar narrowing of the energy gap. This difference in the absorption wavelength can also be observed in the color of the THF solution, as shown in Figure 7(a). Although difficult to observe in the photographs, the CuPc(CN)_8_ THF solution exhibited a slightly greenish color, as compared to that of CuPc.

Figure 7(b) shows the simulated UV–vis spectra of CuPc(CN)_8_ and CuPc. The simulation reproduced the S- and Q-bands, which qualitatively explained the measured results. The simulated absorption spectrum of CuPc(CN)_8_, as compared to that of CuPc, was red-shifted. Introducing eight cyano groups into CuPc to form CuPc(CN)_8_ affected the LUMO more than the SOMO, resulting in a lower LUMO energy and a narrower energy gap, which explains the red shifting of the CuPc(CN)_8_ absorption spectrum. As shown in Figure 7(b), the Q-band transition for both CuPc(CN)_8_ and CuPc is a π → π* transition from SOMO to LUMO with α and β spins. The Q-band absorption wavelengths of CuPc(CN)_8_ and CuPc, calculated using DFT, were 620.01 and 596.35 nm, respectively. The absorption wavelength difference between CuPc(CN)_8_ and CuPc was well reproduced, although the values differed because the DFT calculations cannot exactly reproduce the energy gap values. As shown in Figure S5, the orbital energies around the energy gap of CuPc(CN)_8_ are considerably lower than those of CuPc because of the cyano groups at the molecular terminals. The SOMO and LUMO wave functions of CuPc shown in Figure 7(b) indicate that LUMO has a larger amplitude at the β-position carbons (indicated by the small arrow in Figure 7(b)) where the cyano group bonds to form CuPc(CN)_8_. Thus, the LUMO, as compared to the SOMO, is more strongly affected by the substitution of hydrogen atoms for cyano groups, and the energy gap is smaller because of the stabilization of the LUMO energy, resulting in a red shift of the absorption peak. The MO diagram in Figure 7(b) shows that the SOMO(a_1u_) of CuPc and CuPc(CN)_8_ are not significantly different, but the LUMO(e_g_) of CuPc and CuPc(CN)_8_ are.

**Figure 8:**
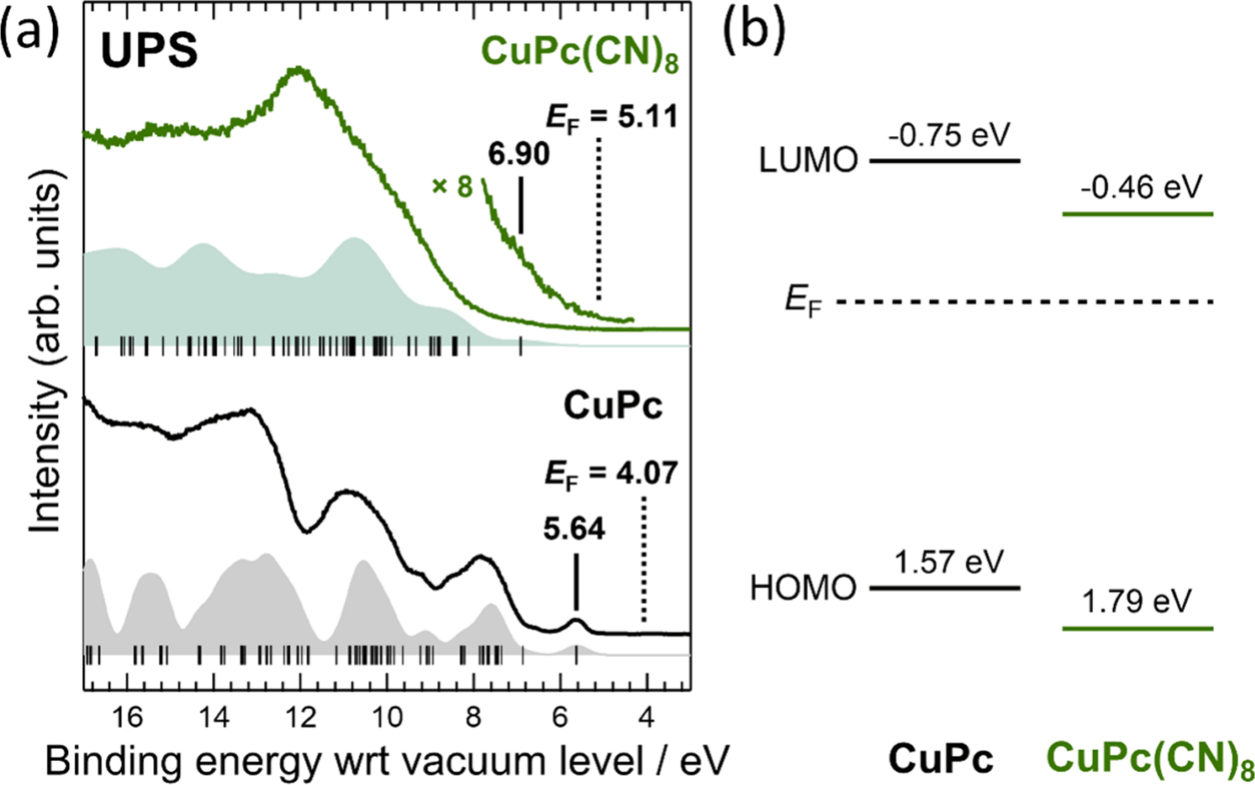
(a) CuPc(CN)_8_ and CuPc UPS results. Solid lines show the measured UPS spectra of CuPc(CN)_8_ and CuPc. The horizontal axis is the binding energy with respect to the vacuum level determined from the secondary electron cutoff of the UPS measurement. The vertical lines at the bottom of each spectrum are the simulated MOs, and the filled spectra are the simulated UPS spectra obtained by convoluting the MOs with the Voigt function. The simulation results are shifted along the horizontal axis so that the calculated the highest occupied molecular orbital (HOMO) positions coincide with the top positions of the experimental HOMO peak. The black vertical lines and numbers shown above the measured spectra indicate the HOMO energies. *E*_F_ denotes the experimentally determined Fermi energy. (b) Energy diagrams of CuPc(CN)_8_ and CuPc measured from the Fermi energy. The HOMO energies were determined using the UPS results and the LUMO energies were estimated by considering the energy gap obtained from the UV–vis absorption edge shown in Figure 7(a). The energy gap was calculated by adding 0.5 eV as the binding energy of the exciton to the observed energy of the absorption edge in the UV–vis spectrum.^33^

UPS provides a replica of the density of states of the occupied states near the Fermi level of the material. Therefore, the UPS spectrum of a molecule provides direct information on the occupied MOs below the HOMO. Figure 8(a) presents the UPS results for CuPc(CN)_8_ and CuPc. The CuPc(CN)_8_ and CuPc UPS spectra correspond well with the simulated spectra. The substitution of hydrogen atoms at the molecular terminals of phthalocyanine with an electron-withdrawing group lowers the frontier orbital energies and is likely to make the molecule an *n*-type molecule.^34^ The HOMO and LUMO energies of F_8_CuPc, F_16_CuPc, and FePc(CN)_8_, as compared to those of CuPc and FePc, were observed at higher binding energies in the UPS spectra.^20,35^ Similar to FePc(CN)_8_, CuPc(CN)_8_ is also expected to be an *n*-type molecule, as compared to CuPc. The HOMO energies of CuPc(CN)_8_ and CuPc were determined from the UPS results to be 6.90 and 5.64 eV, respectively, as measured from the vacuum level. The ionization energy of CuPc(CN)_8_ was approximately 1 eV higher than that of CuPc. The shift in the HOMO energy due to the addition of cyano groups was almost the same as the change in FePc(CN)_8_ with respect to FePc.

Figure 8(b) shows the CuPc(CN)_8_ and CuPc energy diagrams with respect to the Fermi level. The typical binding energy of the exciton in organic semiconductors is approximately one-quarter of the transport gap obtained from UPS and inverse photoemission spectroscopy (IPES) (UPS/IPES) measurements.^33^ Previous studies reported that the UPS/IPES-measured transport gap of CuPc is 2.04 eV; therefore, the binding energy of the exciton in CuPc can be calculated as 0.5 eV.^35^ The energy gaps of CuPc(CN)_8_ and CuPc were obtained from the absorption edge of the UV–vis spectrum shown in Figure 7(a) with the addition of 0.5 eV. Figure 8(b) shows the energy diagrams using the HOMO value obtained from the UPS measurements.

Although CuPc is generally considered to exhibit *p*-type electrical properties, Figure 8(b) shows that the LUMO of CuPc is closer to the Fermi level than to the HOMO. The HOMO, LUMO, and *E*_F_ of CuPc vacuum-deposited films reported in previous studies reporting UPS/IPES results are 5.20, 3.16, and 4.13 eV, respectively, as measured from the vacuum level, with the LUMO being closer to the Fermi level.^35^ Consequently, the HOMO of CuPc in previous studies that measured UPS is approximately 1.5 eV with respect to the Fermi level,^36^ which is consistent with the present results.

Because CuPc is a *p*-type semiconductor, holes are easily injected into the HOMO of CuPc from the Fermi level of the electrode. However, electrons are more easily injected in CuPc(CN)_8_ because the LUMO of CuPc(CN)_8_ is closer to the Fermi level than that of CuPc, which should have n-type electrical properties.

### 3.4 Electrical Properties of CuPc(CN)_8_

**Figure 9:**
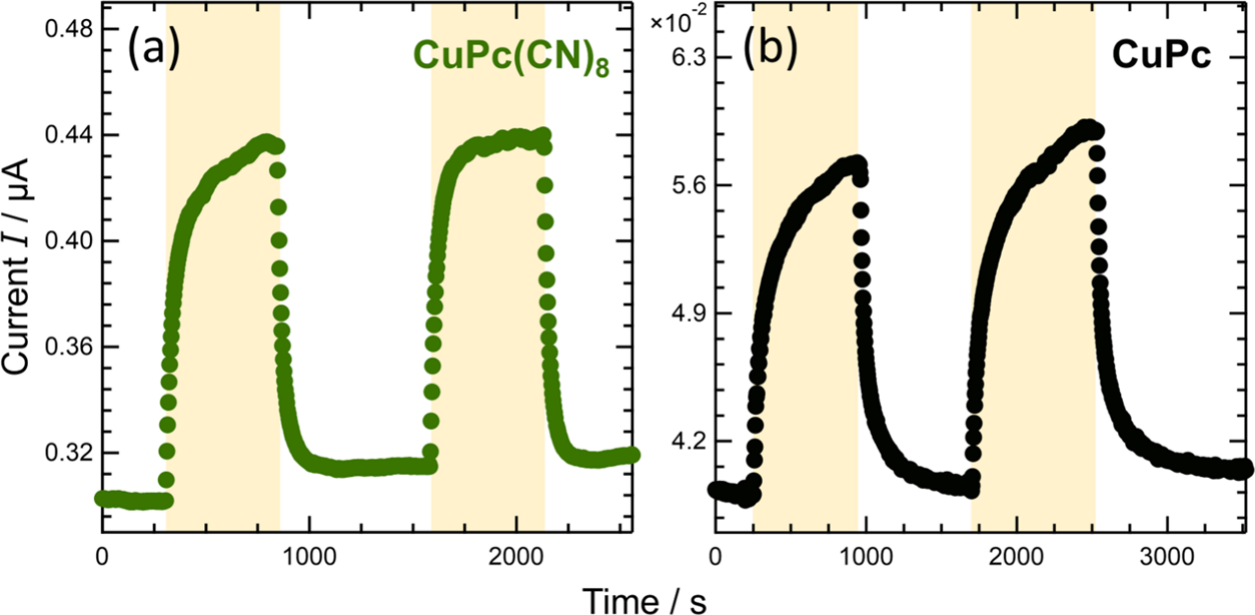
Photocurrent measurement results for (a) CuPc(CN)_8_ and (b) CuPc. Pellet samples were used for the measurements. The applied voltage during the measurement was 3 V. The yellow bands represent the time period of white light irradiation. Figure S6 shows a schematic drawing of the measurement system. Figure S7 shows the LED spectra used in the measurements.

MPcs are well-known organic photoconductors. Phthalocyanine-based molecules, as compared with other photoconductive materials, are currently employed as photoconductors in laser printers as photoelectric conversion materials owing to their superior sensitivity in the wavelength range of 780–800 nm, which is the emission wavelength of semiconductor lasers.^37−39^Figure 9(a),9(b) show the measured light-irradiation dependence of the electrical current of CuPc(CN)_8_ and CuPc, respectively. To perform the electrical measurements, CuPc(CN)_8_ and CuPc powder samples were formed into approximately 1 cm-diameter pellets and placed between an indium tin oxide substrate (positive side) and a copper plate (negative side). As shown in Figure 9, both CuPc(CN)_8_ and CuPc exhibited a rapid increase in current upon light irradiation, which indicates that CuPc(CN)_8_ exhibits photoconductivity similar to that of CuPc. Both CuPc(CN)_8_ and CuPc required in excess of 300 s from the start of light irradiation to saturation of the current value, and from the end of light irradiation to the point where the current decreased to the value before light irradiation. FePc(CN)_8_ and FePc exhibited the same behavior,^20^ which was attributed to carrier traps existing inside the pellet sample and/or at the interface between the sample and electrodes. The increase in the current for CuPc(CN)_8_ and CuPc due to light irradiation was approximately 142 and 148%, respectively, with CuPc exhibiting a slightly larger increase. This trend is similar to that for FePc(CN)_8_ and FePc because the absorption spectrum of CuPc overlaps more with the LED white light spectrum used in the measurement, resulting in the formation of more excitons by white light irradiation and an increase in the number of carriers by charge separation.^20^ However, CuPc(CN)_8_ and CuPc exhibited a lower difference in the increase in current owing to light irradiation than FePc(CN)_8_ (∼129%) and FePc (∼150%). This may be due to the smaller difference in the wavelength range of the absorption peaks of CuPc and CuPc(CN)_8_ than that of FePc and FePc(CN)_8_.

**Figure 10:**
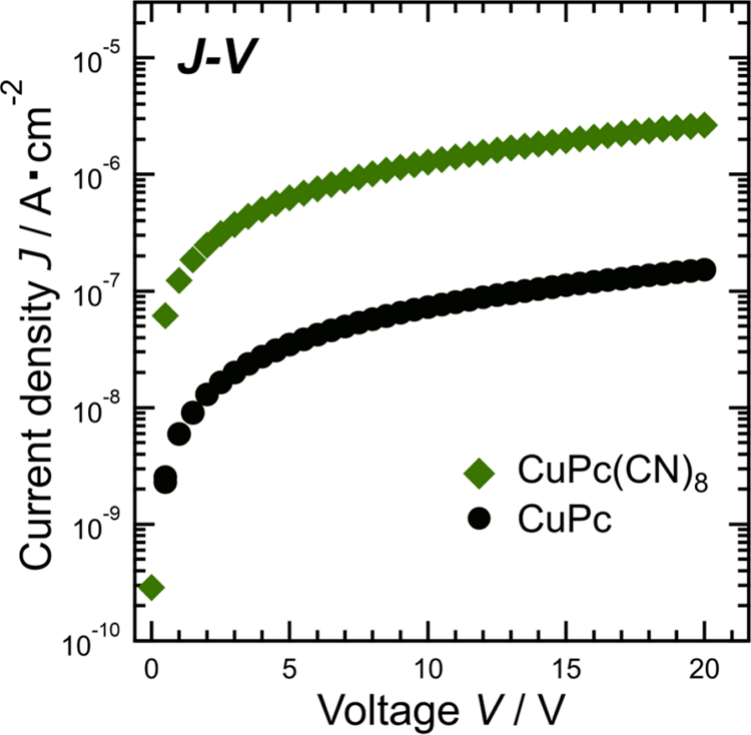
Voltage dependence of the current density: current density–voltage (*J–V*) characteristics of CuPc(CN)_8_ and CuPc. Powder samples processed into pellets were used as the samples. The vertical axis is on the logarithmic scale.

Figure 10 shows the *J–V* plots for CuPc(CN)_8_ and CuPc. Figure S8 shows the *J–V* characteristics with the vertical axis on a linear scale. The electrical conductivities of CuPc(CN)_8_ and CuPc in the low-voltage range where *J* can be fitted with a straight line were 3.42 ± 0.03 and 0.19 ± 0.01 nS/cm, respectively. The conductivity of CuPc(CN)_8_ was approximately 18-fold higher than that of CuPc, but lower than that of FePc(CN)_8_ (4.78 ± 0.10 nS/cm).^20^ The enhanced conductivity of FePc(CN)_8_ can be explained by the large overlap between π-orbitals due to its x-form-like crystal structure. However, CuPc(CN)_8_ does not have a crystal structure with a particularly large overlap of π-orbitals, as compared to CuPc; therefore, the crystal structure is not related to the conductivity improvement. The improved conductivity of CuPc(CN)_8_ can be attributed to a reduction in the charge injection barrier from the electrode, as shown in Figure 8(b). The graph in Figure S8 shows that CuPc(CN)_8_ exhibits ohmic behavior over a wide range of positive and negative voltages, indicating ambipolar conduction.

### 3.5 Comparison of CuPc(CN)_8_ and FePc(CN)_8_

The basic properties of the CuPc(CN)_8_ molecule are similar to those of FePc(CN)_8_. Both have cyano groups at the molecular terminals, which results in smaller energy gap and lower frontier orbital’s energy, making them *n*-type. CuPc(CN)_8_ and FePc(CN)_8_ are also similar in that they exhibit higher electrical conductivity than CuPc and FePc. On the other hand, the crystal structures of FePc(CN)_8_ and CuPc(CN)_8_ are very different as discussed in Section 3.1. Interestingly, FePc(CN)_8_ and CuPc(CN)_8_ have in common that molecular layers are formed by intermolecular hydrogen bonds, but the stacking manner of the molecular layers is different. This may influence the difference in their thermal stability, as revealed by TG-DTA measurements, as discussed in Section 3.2.

## 4 Conclusions

This article reports the facile synthesis and characterization of CuPc(CN)_8_ powder. Intermolecular hydrogen bonds play an important role in the formation of the crystal structure of CuPc(CN)_8_: the intermolecular hydrogen bonds formed between the nitrogen of the cyano group at the molecular terminal of CuPc(CN)_8_ and the hydrogen of the benzene ring of the neighboring molecule play an important role in the formation of molecular layers parallel to the molecular plane. This hydrogen bond does not have a counterpart hydrogen in the direction of the CN bond axis of the cyano group. These intermolecular hydrogen bonds are similar to those in FePc(CN)_8_, but the CuPc(CN)_8_ crystal structure differs from that of FePc(CN)_8_. The space group of the CuPc(CN)_8_ crystal is P1̅, which differs from that of the FePc(CN)_8_ crystal structure similar to x-form, which is classified in the *P*4/*mcc* space group. A previous study reported that FePc(CN)_8_ synthesized using thermal polymerization contains impurities resulting from the hydrolysis of cyano groups at the molecular terminals during the synthesis process;^20^ however, the FTIR and XPS analyses confirmed that the CuPc(CN)_8_ synthesized in this study contained few impurities. The thermal stability of the samples was also examined using TG-DTA, which revealed that the thermal decomposition temperature of CuPc(CN)_8_ is approximately 420 °C, which is considerably higher than that of FePc(CN)_8_ (∼260 °C). A comparison of the observed and simulated UV–vis results showed that the electronic structure of CuPc(CN)_8_, as compared with that of CuPc, is considerably influenced by the cyano groups at the molecular terminals, which lower the LUMO energy, resulting in a narrowing of the energy gap, leading to a red-shift of the absorption spectrum by approximately 20 nm. The UPS spectrum of CuPc(CN)_8_ corresponded well with that obtained from the MO calculations. The UPS results showed that the CuPc(CN)_8_ ionization energy was approximately 1 eV larger than that of CuPc, suggesting enhanced *n*-type electrical properties, as compared with CuPc. CuPc(CN)_8_ exhibited the photoconduction characteristics of phthalocyanines with an electrical conductivity approximately 18-fold higher than that of CuPc, which was determined from the UPS and UV–vis results and is attributed to the LUMO of CuPc(CN)_8_ being closer to the Fermi level and the reduction of the electron injection barrier from the electrode. CuPc(CN)_8_ is expected to be applied as a new *n*-type organic semiconductor owing to its high electrical conductivity and thermal stability. Furthermore, the CuPc(CN)_8_ synthesis method reported in this study will greatly contribute to the realization of CuPc-MOF, which is expected to realize magnetic ordering and interesting electronic structures, such as Dirac and flat bands.

## Supporting Information Available

Atomic coordinates of CuPc(CN)_8_ crystals determined from XRD simulations; results of the FTIR measurements of CuPc and FTIR simulations of CuPc based on vibrational calculations; results of XPS survey scans of CuPc(CN)_8_ and CuPc; O 1s XPS spectrum of CuPc(CN)_8_; peak positions of C 1s, N 1s, Cu 2p_3/2_, and O 1s XPS spectra determined by the fitting analysis; FTIR spectrum of CuPc(CN)_8_ after heating at 470 °C for 24 h at 1.9 × 10^2^ Pa in a nitrogen atmosphere; results of the MO calculations of CuPc(CN)_8_ and CuPc using DFT; schematic diagram of the measurement system used for the electrical measurements; spectrum of the white LED used to measure the light irradiation dependence of the photoconductivity; and *J–V* graph of CuPc(CN)_8_ and CuPc with the vertical axis on a linear scale (PDF)
