# Correction to “Synthesis and Characterization of Octacyano-Cu-Phthalocyanine”

**DOI:** 10.1021/acsomega.4c07840

**Published:** 2024-09-18

**Authors:** Momoka Isobe, Fumiya Abe, Shunsuke Takagi, Kaname Kanai

In the original article, there were mistakes in Figure 2(a) and unit cell parameters for the crystal structure of CuPc(CN)_8_.

**Figure 2:**
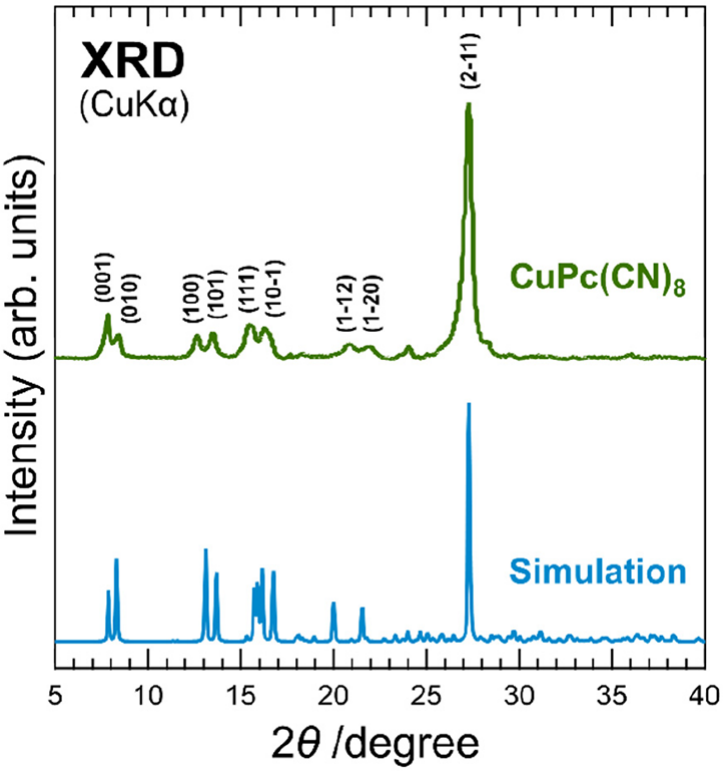
(a) XRD patterns of CuPc(CN)_8_. The lower part of the graph shows the simulated diffraction pattern.

Figure 2(a) contains mistakes in the Miller indexes for each peak. With the correction to Figure 2(a), (1–10) and (11–1) in the text need to be corrected to (001) and (010), respectively (in the left column on page 32135).

Unit cell parameters in the right column on page 32135 of the text and in the caption of Table S1 (Supporting Information) should correctly be as follows. Unit cell parameters: *a* = 0.69395 nm, *b* = 1.0650 nm, and *c* = 1.1539 nm, α = 90.7498°, β = 77.0608°, and γ = 88.6037°.

