# Supplementary material for: Synthesis and Characterization of Octacyano-Cu-Phthalocyanine
Source: ACS Omega. 2024 Jul 11;9(29):32133–43. doi: 10.1021/acsomega.4c04292 (PMC11270545; doi:10.1021/acsomega.4c04292)
Supplement: Supplementary file 1 — ao4c04292_si_001.pdf [file ao4c04292_si_001.pdf]

Supporting Information

Synthesis and characterization of octacyano-Cu-phthalocyanine

Momoka Isobe, Fumiya Abe, Shunsuke Takagi, Kaname Kanai*

Department of Physics and Astronomy, Faculty of Science and Technology, Tokyo University of Science, 2641 Yamazaki, Noda, Chiba 278-8510, Japan.

Experimental

Fourier transform infrared (FTIR) spectra for samples embedded in KBr pellets were acquired using a spectrometer (JASCO Corporation, FTIR-6100).

X-ray photoemission spectroscopy (XPS) (JPS-9030/JEOL Ltd.) measurements were performed using Al Kα radiation (λ = 1486.6 eV) as the excitation source. The XPS profiles presented in this study were analyzed using Voigt functions with the XPSPEAK41 software (written by Raymund W. M. Kwok).

The PL spectrum of the LED was measured using a BIM-6002A-10 spectrometer (Brolight Technology Co., Ltd.) connected to an optical fiber.

Electric current measurements were performed using a source-measure unit (6487 J, Keithley) and DC power source (R6144, Advantest).

Theoretical

The FTIR simulations were performed for a single molecule using Gaussian09 (B3LYP/6-31G(d)). Molecular Orbital (MO) calculations for the isolated molecules based on DFT calculations were performed using Gaussian09 (B3LYP/6–31G(d)). Theoretical calculations for CuPc and CuPc$(CN)_8$ were performed for their S = 1/2 doublet ground states.

Table S1. Atomic coordinates of $CuPc(CN)_8$ crystal determined by the XRD simulation.

Unit cell parameters: a = 7.1477 nm, b = 10.663 nm, c = 11.555 nm, α = 90.3353°, β = 76.7172°, and γ = 86.8458°.

Atom	*a*	*b*	*c*
C1	0.64614	1.12043	0.34990
N2	0.61368	0.55061	-0.16327
C3	0.79169	1.19475	0.11265
N4	0.55343	0.66483	0.06055
C5	0.88673	0.59981	-0.69079
C6	0.97913	0.83262	-0.59864
C7	0.35485	-0.03384	-0.25521
C8	0.27490	-0.07033	-0.13570
C9	0.48662	0.29724	-0.17769
C10	0.35841	0.23958	0.00814
C11	0.42368	0.16807	-0.18509
C12	0.34246	0.13247	-0.06770
C13	0.43174	0.08581	-0.28045
C14	0.26777	0.01398	-0.04081
N15	0.64820	1.19156	0.42757
N16	0.84105	1.29789	0.09284
C17	0.88797	0.73869	-0.51969
C18	0.83976	0.62104	-0.56565
C19	0.69122	0.66552	-0.19467
C20	0.62244	0.47974	-0.26419
C21	0.75585	0.66975	-0.32329
C22	0.70954	0.55341	-0.36788
C23	0.84527	0.76360	-0.39706
C24	0.74999	0.52730	-0.48944
N25	0.43376	0.63749	0.27252
N26	0.07274	0.41495	0.79403
N27	-0.05059	0.08814	0.66192
H29	0.20712	-0.01303	0.05080

H30	0.49513	0.11300	-0.37169
H31	0.88083	0.85370	-0.36185
H32	0.71527	0.43698	-0.52470
N33	0.70595	0.76262	-0.12513
Cu	0.50000	0.50000	0.00000

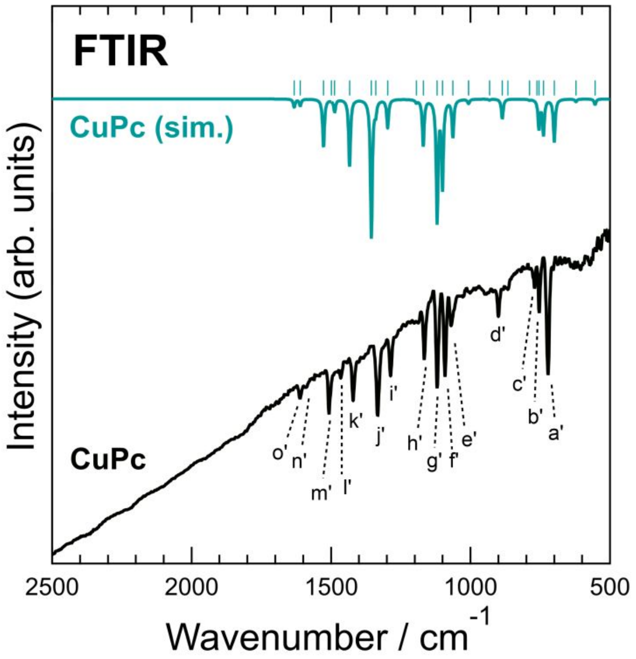

Figure S1. FTIR spectra of CuPc. The labels on each peak correspond to the vibrations listed in Table 2. The upper part of the graph shows the simulated FTIR results for a single molecule of CuPc. The simulated spectra were shifted by approximately -31 cm^{-1} to reproduce the peak positions in the fingerprint region of the measurements.

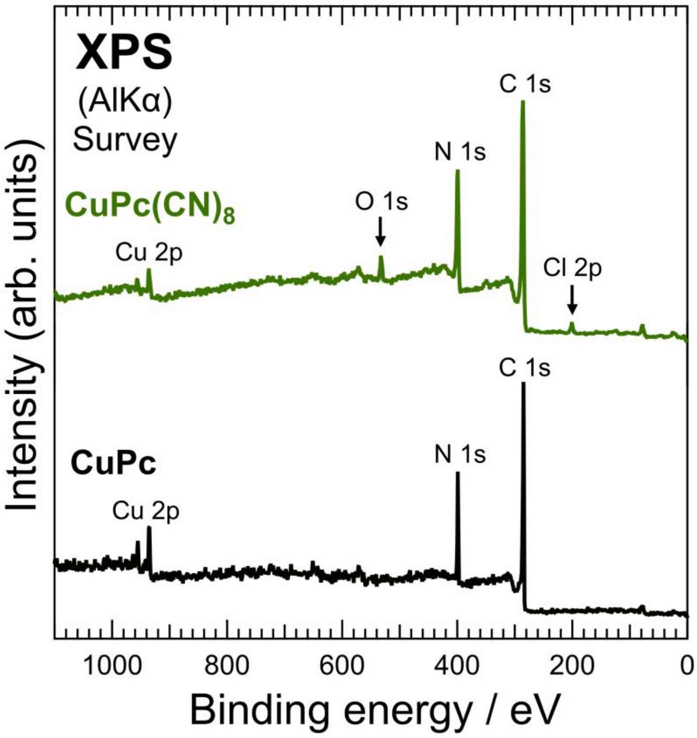

Figure S2. XPS survey scan of $CuPc(CN)_8$ and CuPc.

The spectrum of $CuPc(CN)_8$ shown in Figure S2 shows a Cl 2p peak around 200 eV, indicating that the $CuPc(CN)_8$ sample contains chlorine that can be regarded as originating from impurities.

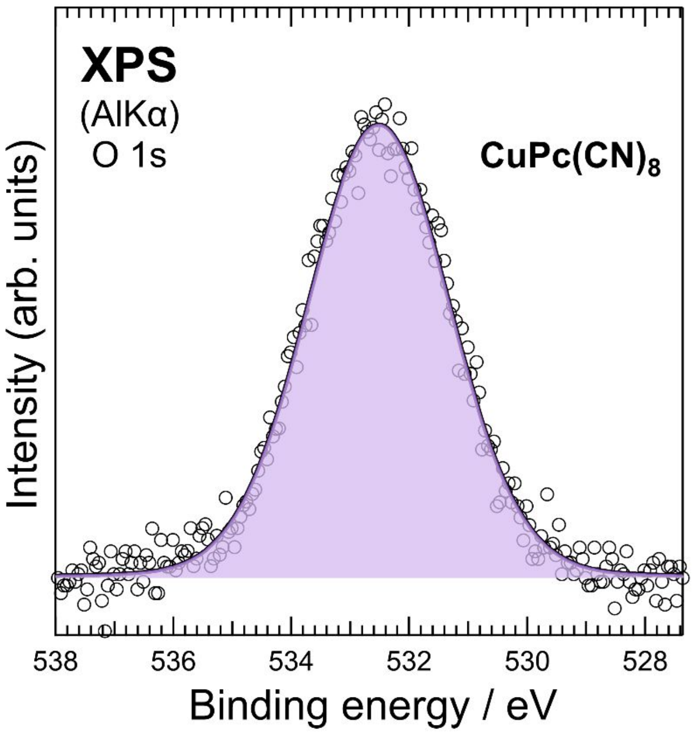

Figure S3. XPS result of the O 1s core level of $CuPc(CN)_8$.

Table S2. Peak positions of the C 1s, N 1s, and Cu $2p_{3/2}$ XPS peaks of $CuPc(CN)_8$ and CuPc in Figure 4(b)-(d) determined from the fitting analysis. Peak position of O 1s XPS peak in Figure S3 is also given. The letters in the left column correspond to the labels assigned to each peak in Figure 4(b)-(d). S represents satellite peak. Unit is in eV.

	$CuPc(CN)_8$	CuPc
C_1	286.6	286.0
C_2	285.6	284.8
C_3	286.6	-
$S(C_1)$	288.6	288.0
$S(C_2)$	287.6	286.8
$S(C_3)$	288.6	-
S(C)-1	290.3	289.0
S(C)-2	292.0	291.0
S(C)-3	-	293.0
N_1	400.1	399.6
N_2	399.2	398.8
N_3	399.2	-
$S(N_1)$	402.1	401.6
$S(N_2)$	401.2	400.8
$S(N_3)$	401.2	-
S(N)-1	403.3	404.6
S(N)-2	404.7	406.2
S(N)-3	405.9	403.4
Cu(0)	933.0	-
Cu^{2+}	935.7	935.8
S(Cu)-1	942.0	941.8
S(Cu)-2	944.2	943.9
S(Cu)-3	945.9	945.8
O 1s	532.5	533.5

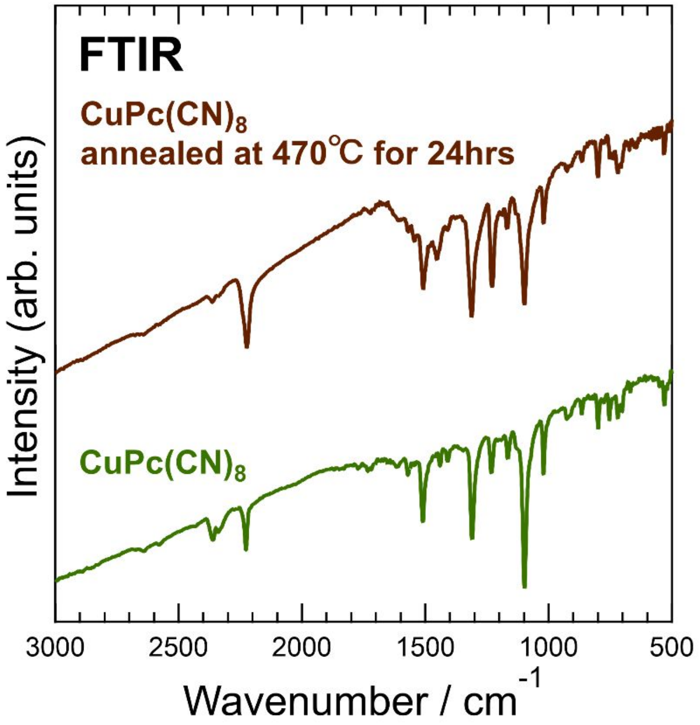

Figure S4. FTIR spectrum of $CuPc(CN)_8$ after heating at 470°C for 24 hours in a nitrogen atmosphere at a pressure of 1.9×10^2 Pa. The lower spectrum is the FTIR spectrum of $CuPc(CN)_8$ before heating.

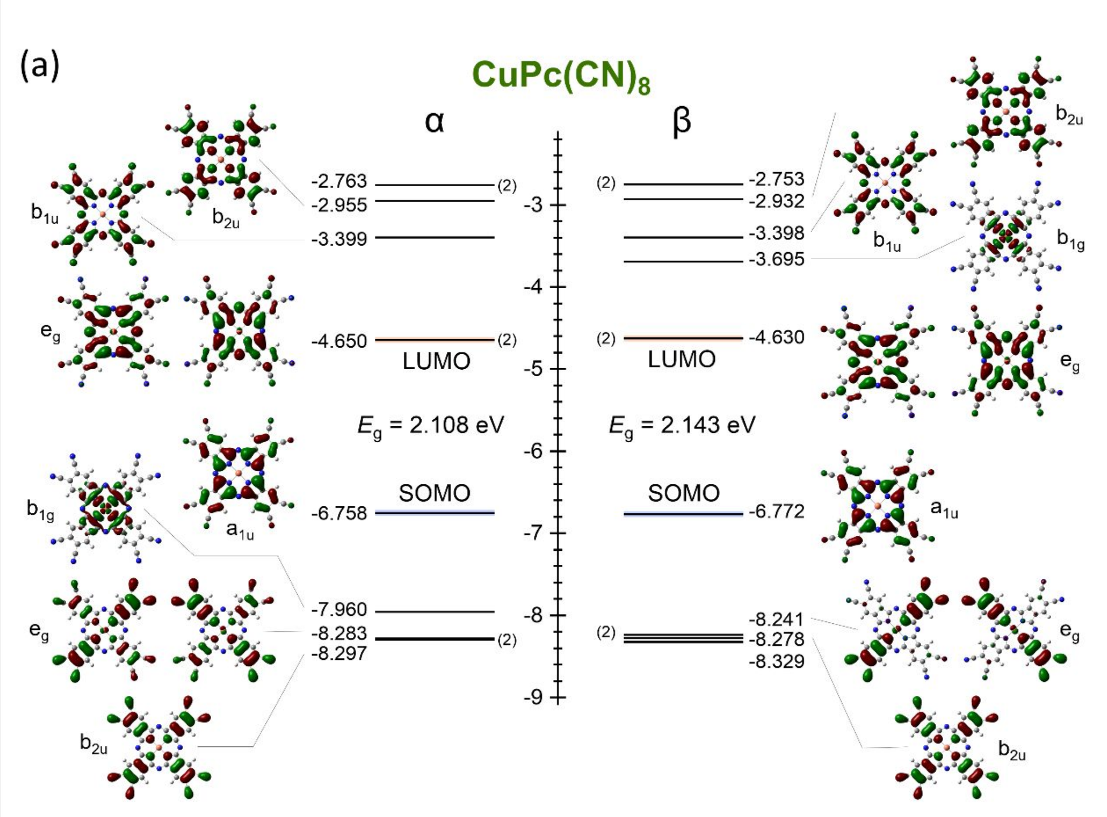
(a)
CuPc(CN)8
α
β
b1u
b2u
-2.763
-2.955
-3.399
(2)
-3
-2.753
-2.932
-3.398
-3.695
b1g
eg
-4.650
LUMO
-4
-5
-4.630
Eg = 2.108 eV
Eg = 2.143 eV
-6
a1u
SOMO
-6.758
-6.772
-7
-7.960
-8.283
-8.297
-8
-8.241
-8.278
-8.329
-9

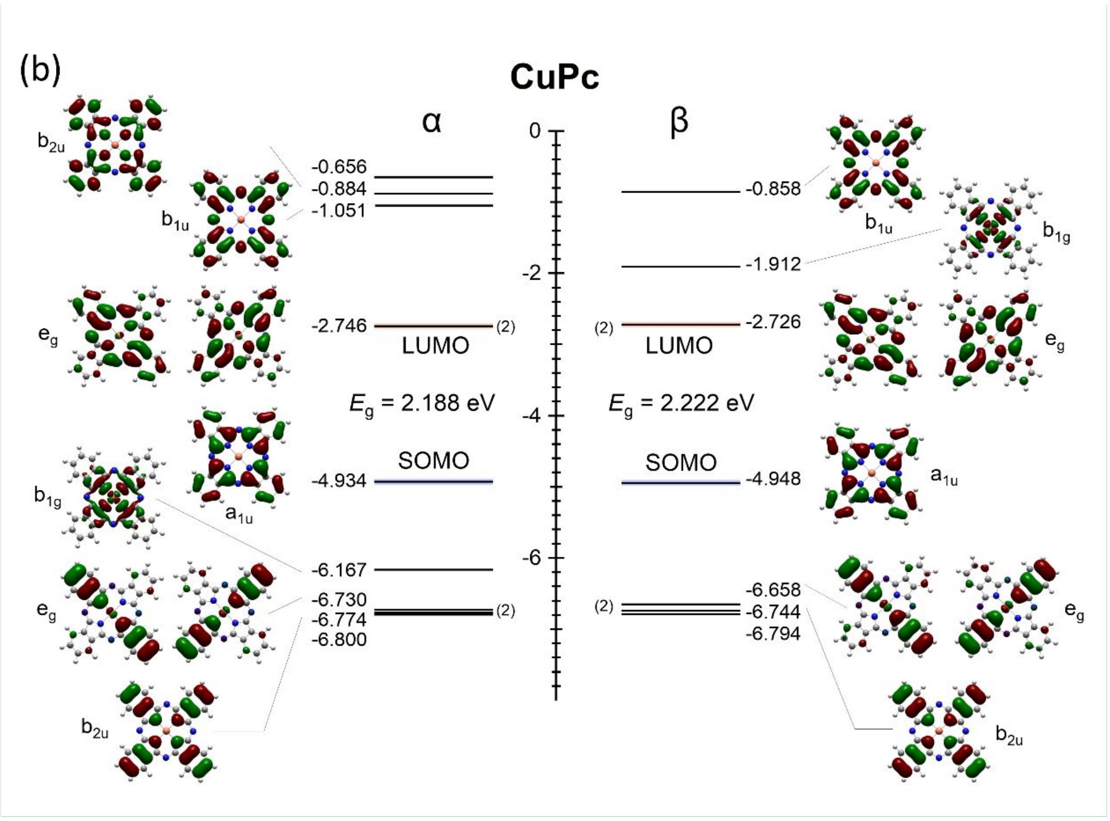
(b)
CuPc
α
β
0
b2u
b1u
-0.656
-0.884
-1.051
-0.858
b1g
-2
-1.912
eg
-2.746
LUMO
(2)
-2.726
Eg = 2.188 eV
Eg = 2.222 eV
-4
SOMO
-4.934
-4.948
a1u
-6
-6.167
-6.730
-6.774
-6.800
-6.658
-6.744
-6.794

Figure S5. The results of the MO calculations based on DFT for (a) $CuPc(CN)_8$ and (b) CuPc. The numbers in the diagram show the energy of each orbital, measured from the vacuum level. The units are in eV. The red and green lobes of the MOs indicate the different signs of the wave functions.

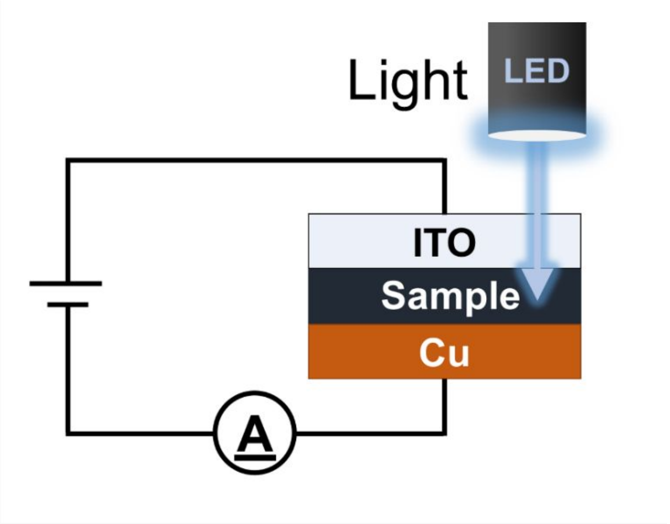

Figure S6. Schematic drawing of the measurement system used for the electrical measurements discussed in Figure 9 and Figure 10.

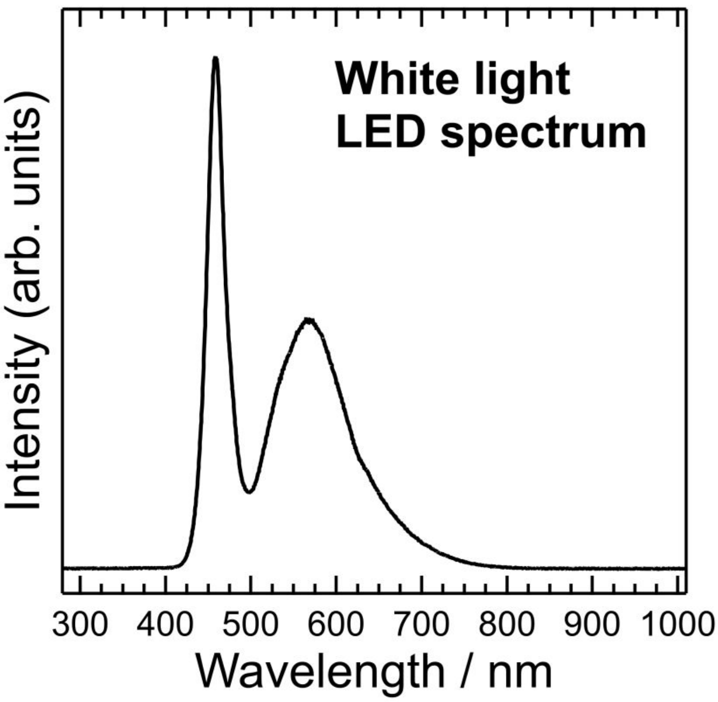

Figure S7. Spectrum of the white-light LED used to measure the electrical characteristics shown in Figure 9.

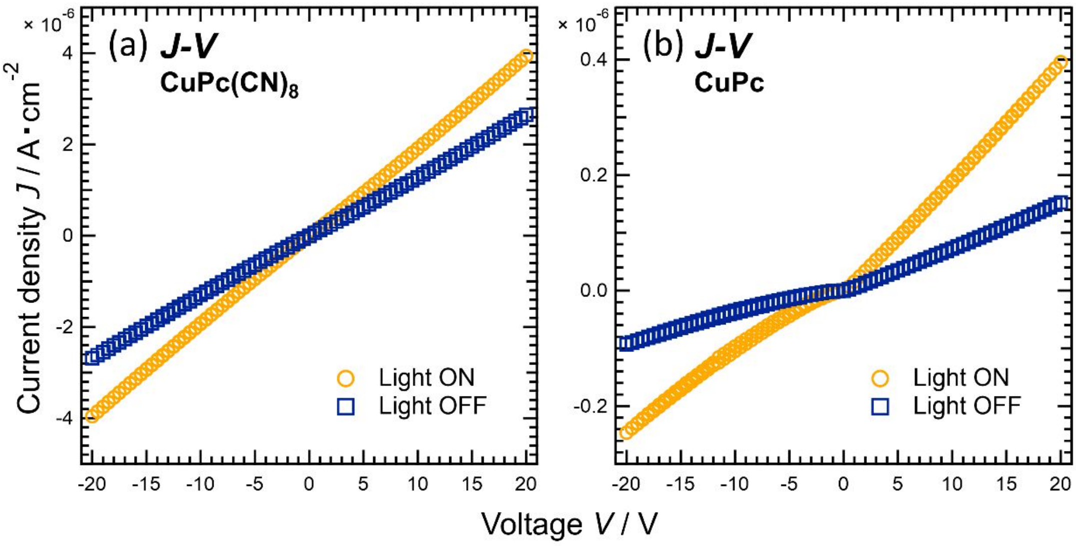

Figure S8. *J-V* characteristics of (a) $CuPc(CN)_8$ and (b) CuPc, with a linear scale on the vertical axis. The data are the same as in Figure 10. Yellow circles are results obtained under LED light irradiation, blue squares are results obtained without the light irradiation.
